# Supplementary material for: Antidiabetic, Antihyperlipidemic, and Antioxidant Evaluation of Phytosteroids from Notholirion thomsonianum (Royle) Stapf
Source: Plants (Basel). 2023 Oct 17;12(20):3591. doi: 10.3390/plants12203591 (PMC10609873; doi:10.3390/plants12203591)
Supplement: Supplementary file 1 [file plants-12-03591-s001.zip › plants-2586016-supplementary.pdf]

Antidiabetic, Anti-hyperlipidemic and Antioxidant Evaluations of Phytosteroids from *Notholirion thomsonianum* (Royle) Stapf

Table S1: GCMS results of compound **NtSt01**.

Compound Label	Name	*m/z*	RT	Algorithm
3.beta.-Acetoxystigmasta-4,6,22-triene	**3.beta.-Acetoxystigmasta-4,6,22-triene**	55.1	56.964	Find by Chromatogram Deconvolution

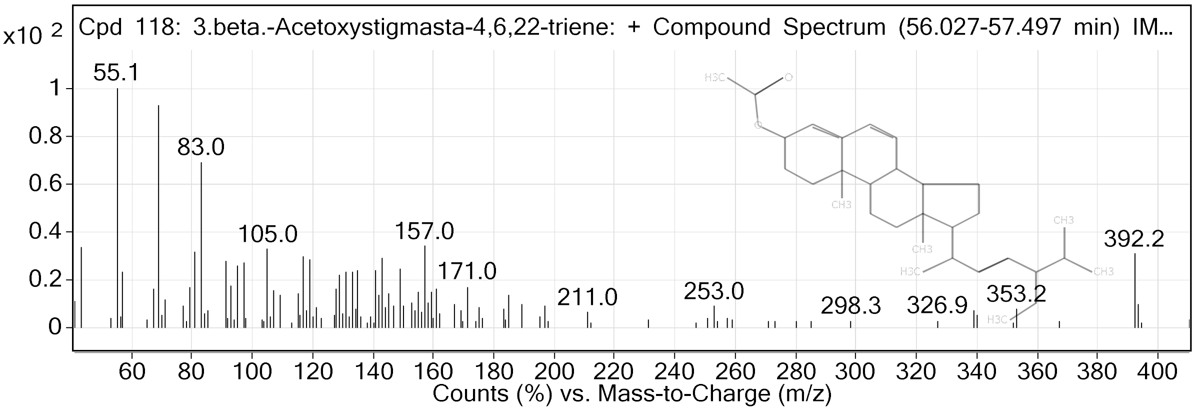

Figure S1: MS spectrum and fragmentation pattern of **NtSt01**.

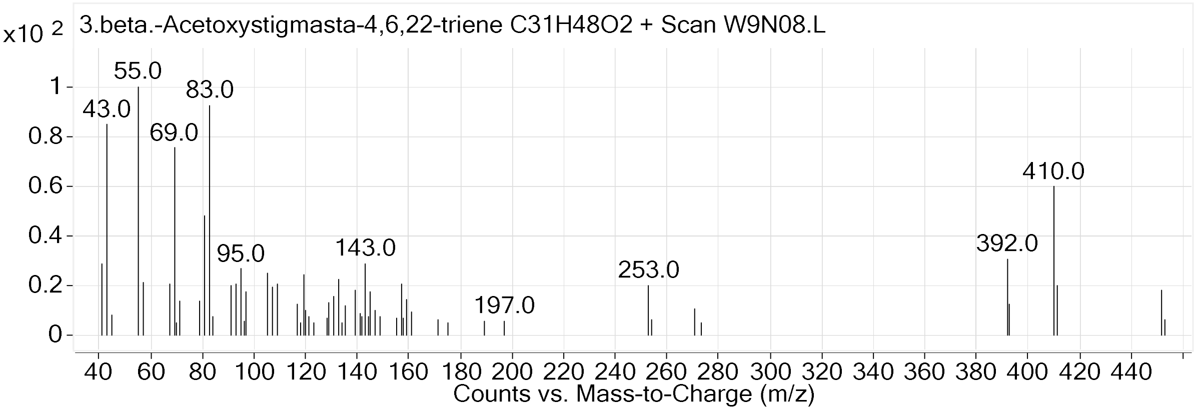

Figure S2: Library spectrum and fragmentation pattern of **NtSt01**.

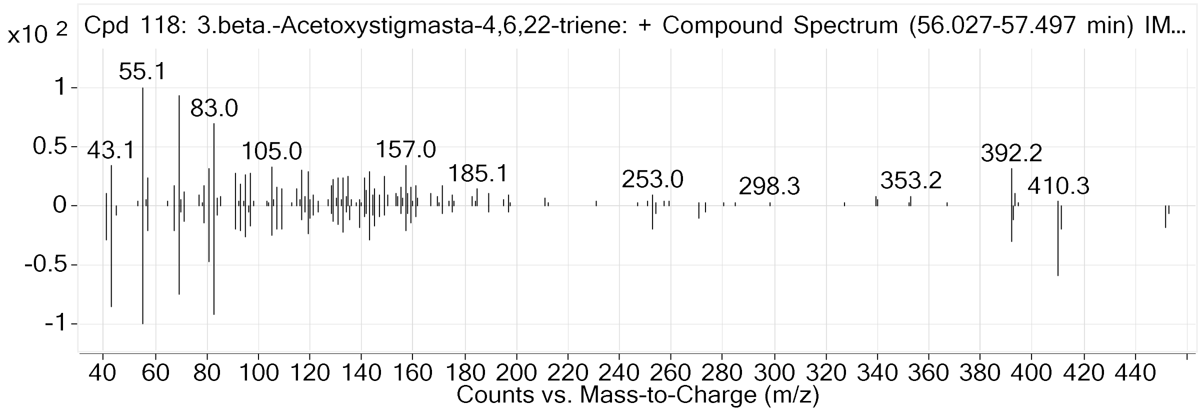

Figure S3: Difference spectrum of **NtSt01**.

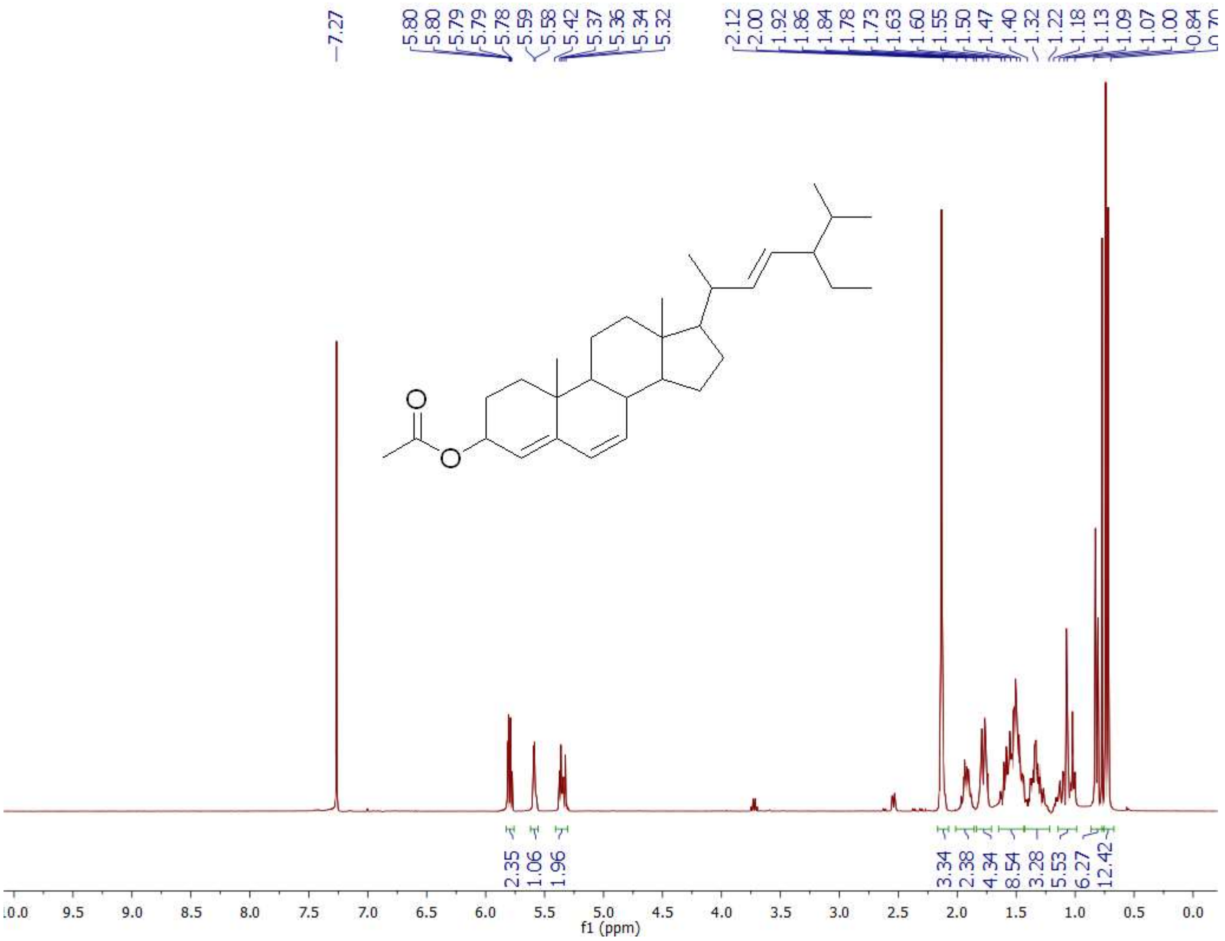

Figure S4: ^{1}H NMR spectrum of **NtSt01**.

Table S1: GCMS results of compound **NtSt02**.

Compound Label	Name	*m/z*	RT	Algorithm
4,6-Cholestadien-3beta-ol, benzoate	**Cholesta-4,6-dien-3-ol, benzoate, (3.beta.)- (CAS)**	135	57.812	Find by Chromatogram Deconvolution

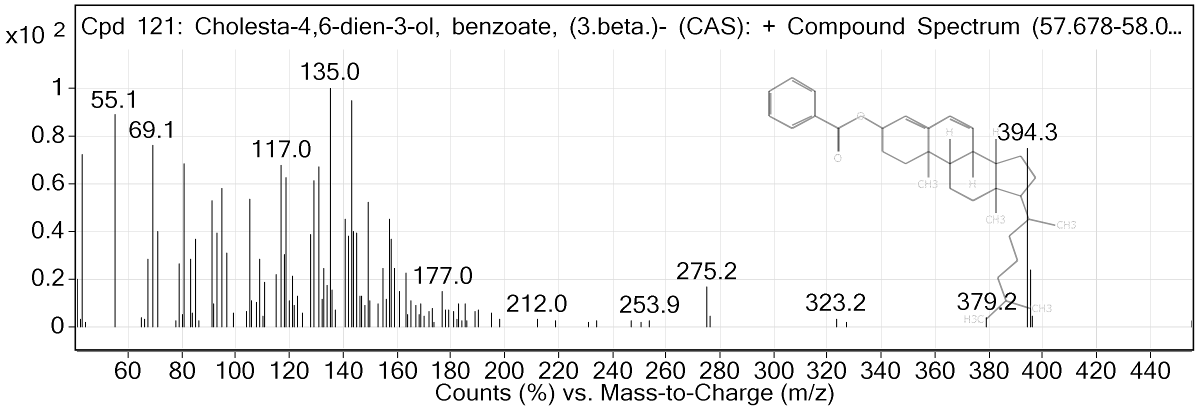

Figure S5: MS spectrum and fragmentation pattern of **NtSt02**.

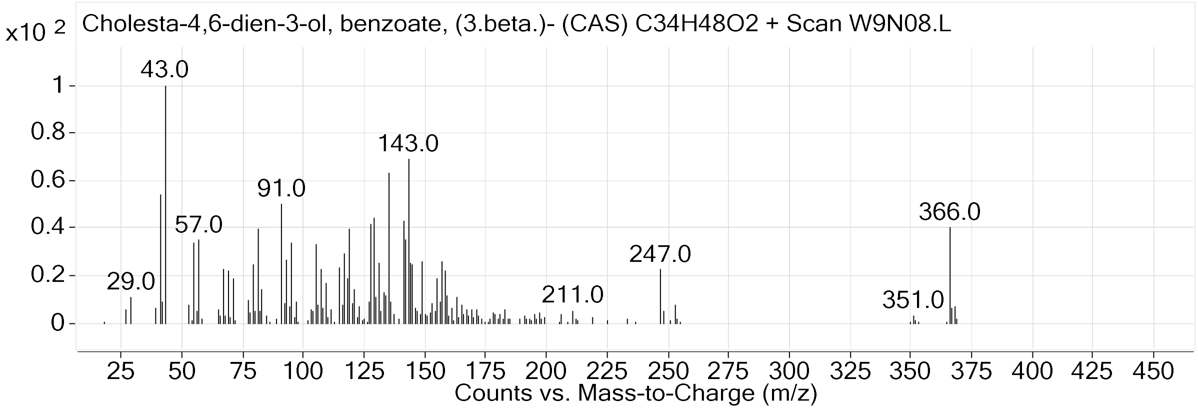

Figure S6: Library spectrum and fragmentation pattern of **NtSt02**.

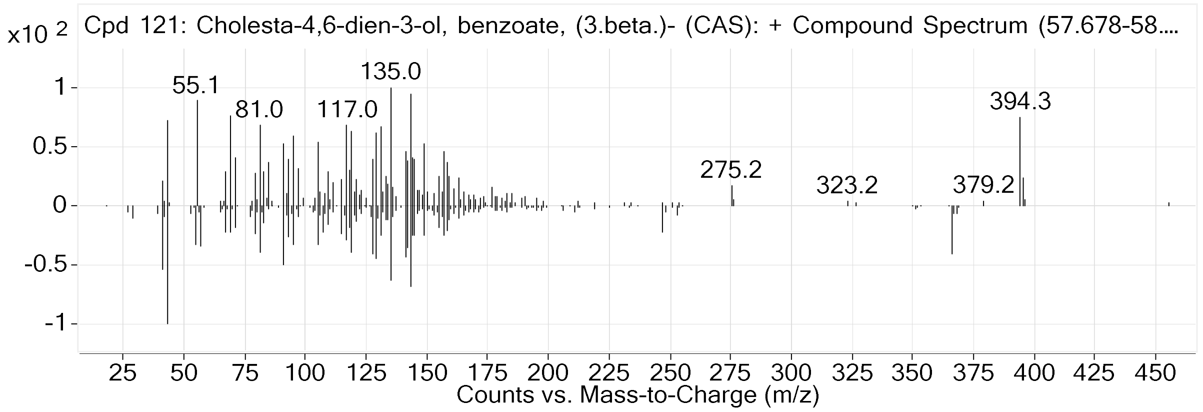

Figure S7: Difference spectrum of **NtSt02**.

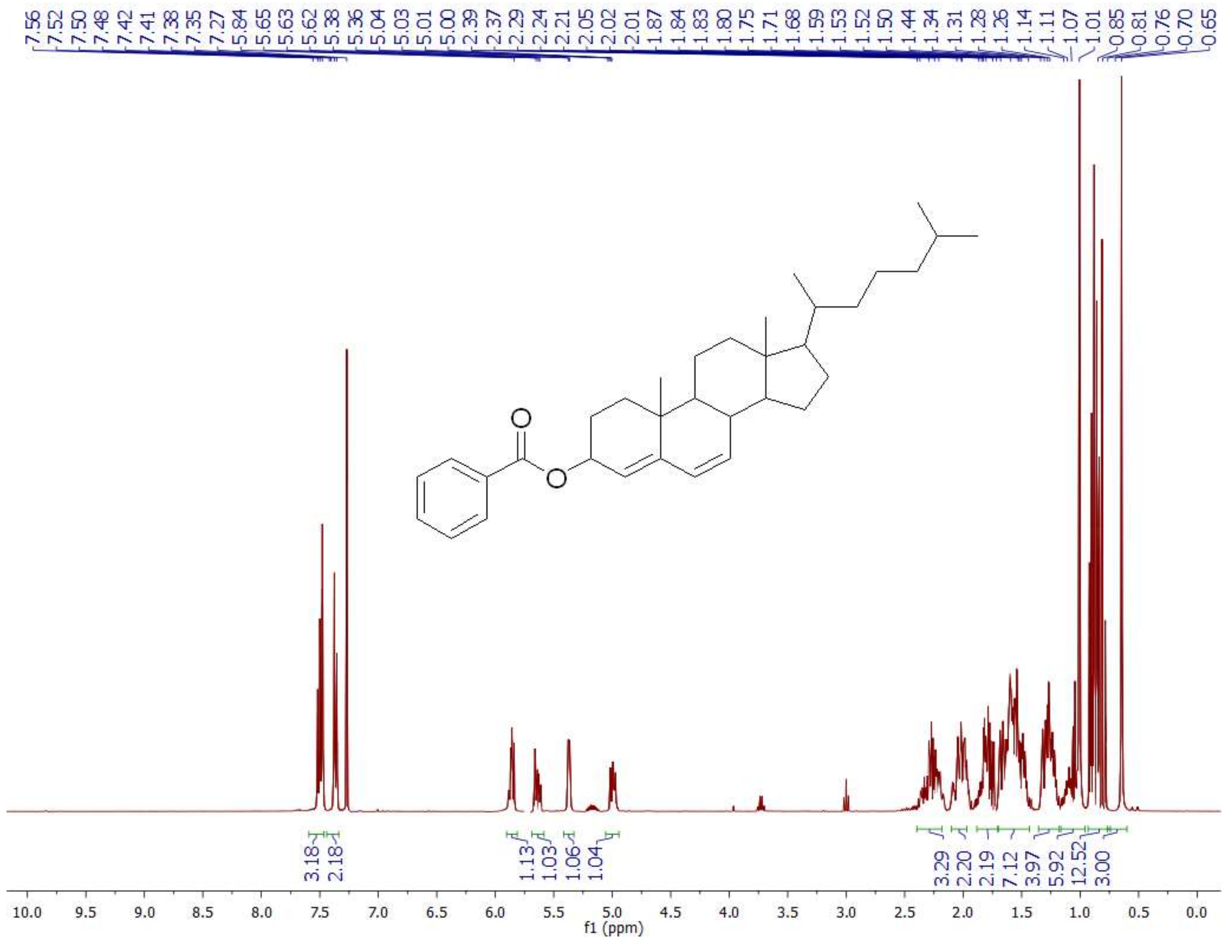

Figure S8: ^{1}H NMR spectrum of **NtSt08**.
